# Supplementary material for: Efficacy and Safety of OnabotulinumtoxinA 400 Units in Patients with Post-Stroke Upper Limb Spasticity: Final Report of a Randomized, Double-Blind, Placebo-Controlled Trial with an Open-Label Extension Phase
Source: Toxins (Basel). 2020 Feb 18;12(2):127. doi: 10.3390/toxins12020127 (PMC7077183; doi:10.3390/toxins12020127)
Supplement: Supplementary file 1 [file toxins-12-00127-s001.pdf]

Supplementary Materials: Efficacy and Safety of OnabotulinumtoxinA 400 Units in Patients with Post−Stroke Upper Limb Spasticity: Final Report of a Randomized, Double−Blind, Placebo−Controlled Trial with an Open−Label Extension Phase

Masahiro Abo, Takashi Shigematsu, Hiroyoshi Hara, Yasuko Matsuda, Akinori Nimura, Yoshiyuki Yamashita and Kaoru Takahashi

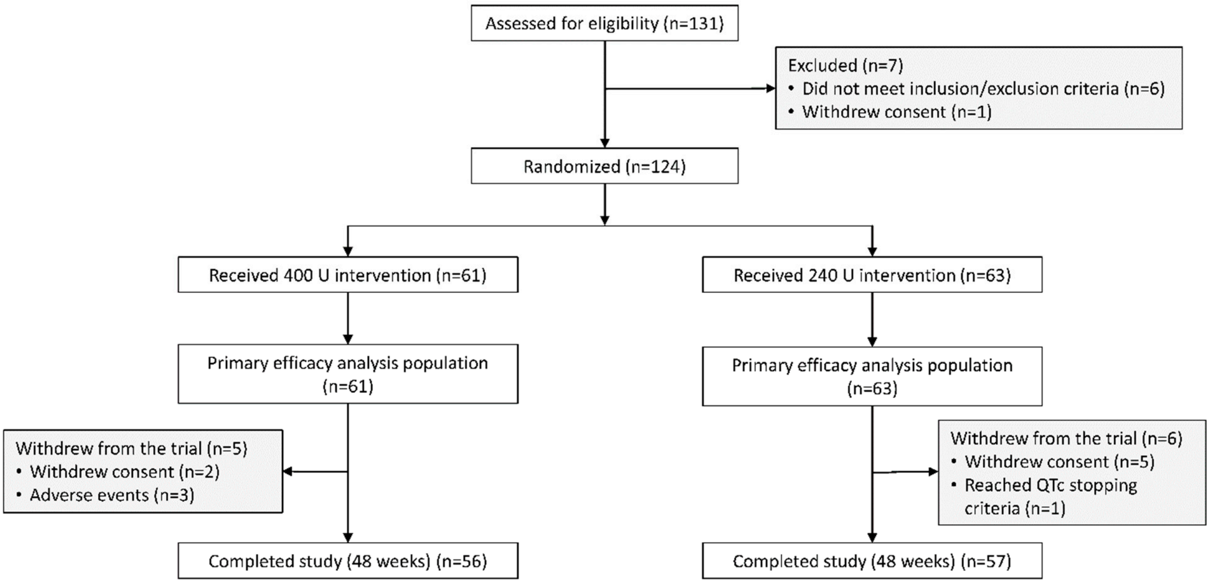

Figure S1. Patient disposition.

Table S1. MAS scores[a] in the double−blind and open−label phases.

	Week 0		Week 2		Week 4		Week 6		Week 12	
	**n**	**Mean (95% CI)**	**n**	**Mean (95% CI)**	**n**	**Mean (95% CI)**	**n**	**Mean (95% CI)**	**n**	**Mean (95% CI)**
**First injection (double−blind phase)**										
400 U group										
Elbow	61	4.1 (4.0, 4.2)	60	3.0 (2.8, 3.2)	59	3.0 (2.7, 3.2)	59	3.0 (2.7, 3.3)	57	3.5 (3.3, 3.7)
Wrist	61	3.8 (3.7, 4.0)	60	2.6 (2.3, 2.9)	59	2.5 (2.2, 2.8)	59	2.5 (2.2, 2.8)	57	3.3 (3.1, 3.5)
Fingers	61	3.7 (3.6, 3.9)	60	2.5 (2.2, 2.8)	59	2.4 (2.1, 2.7)	59	2.5 (2.3, 2.8)	57	3.2 (3.0, 3.5)
Thumb	54	3.4 (3.2, 3.6)	53	2.4 (2.1, 2.7)	53	2.2 (1.9, 2.6)	53	2.3 (2.0, 2.6)	51	2.8 (2.5, 3.1)
240 U group										
Elbow	63	4.1 (4.0, 4.2)	63	3.5 (3.3, 3.7)	63	3.4 (3.2, 3.6)	63	3.4 (3.2, 3.6)	60	3.8 (3.6, 3.9)
Wrist	63	3.8 (3.6, 4.0)	63	2.5 (2.2, 2.8)	63	2.3 (2.0, 2.6)	63	2.5 (2.2, 2.7)	60	3.1 (2.8, 3.4)
Fingers	63	3.8 (3.6, 4.0)	63	2.4 (2.2, 2.7)	63	2.3 (2.1, 2.6)	63	2.4 (2.2, 2.7)	60	3.2 (2.9, 3.4)
Thumb	60	3.4 (3.1, 3.6)	60	2.2 (1.9, 2.4)	60	2.1 (1.8, 2.4)	60	2.3 (2.0, 2.6)	57	2.8 (2.5, 3.1)
**Second injection (open−label phase)**										
All patients										

Elbow	116	3.7 (3.6, 3.9)	115	2.7 (2.5, 2.9)	113	2.6 (2.4, 2.8)	115	2.8 (2.6, 3.0)	113	3.3 (3.2, 3.5)
Wrist	105	3.4 (3.2, 3.5)	104	2.4 (2.1, 2.6)	102	2.2 (2.0, 2.4)	104	2.3 (2.1, 2.5)	102	3.0 (2.8, 3.2)
Fingers	111	3.3 (3.2, 3.5)	110	2.5 (2.2, 2.7)	108	2.4 (2.2, 2.6)	110	2.5 (2.3, 2.7)	108	3.2 (3.0, 3.3)
Thumb	94	3.0 (2.8, 3.2)	94	2.1 (1.9, 2.3)	92	2.1 (1.9, 2.3)	93	2.2 (2.0, 2.4)	92	2.7 (2.5, 2.9)
Shoulder[b]	73	3.4 (3.2, 3.6)	73	2.7 (2.5, 2.9)	71	2.6 (2.4, 2.8)	72	2.7 (2.5, 2.9)	71	3.2 (3.0, 3.4)
Forearm pronation[b]	63	3.5 (3.3, 3.7)	62	2.5 (2.3, 2.7)	62	2.6 (2.4, 2.9)	63	2.7 (2.4, 2.9)	61	3.2 (2.9, 3.4)
**Third injection (open−label phase)**										
All patients										
Elbow	109	3.6 (3.5, 3.7)	109	2.7 (2.4, 2.9)	108	2.7 (2.5, 2.9)	108	2.8 (2.6, 3.0)	108	3.2 (3.0, 3.3)
Wrist	95	3.3 (3.1, 3.4)	95	2.3 (2.1, 2.6)	94	2.3 (2.1, 2.5)	95	2.4 (2.2, 2.6)	94	3.0 (2.7, 3.2)
Fingers	103	3.4 (3.2, 3.5)	103	2.5 (2.3, 2.6)	102	2.4 (2.2, 2.6)	103	2.6 (2.4, 2.8)	102	3.2 (3.1, 3.4)
Thumb	76	3.0 (2.7, 3.2)	76	2.2 (1.9, 2.5)	75	2.2 (2.0, 2.4)	76	2.3 (2.0, 2.5)	76	2.8 (2.5, 3.0)
Shoulder[b]	77	3.3 (3.1, 3.4)	77	2.6 (2.4, 2.8)	77	2.6 (2.4, 2.8)	76	2.7 (2.5, 2.9)	76	3.0 (2.8, 3.2)
Forearm pronation[b]	66	3.3 (3.0, 3.5)	66	2.5 (2.2, 2.7)	66	2.5 (2.3, 2.8)	66	2.7 (2.4, 2.9)	65	2.9 (2.7, 3.2)
**Fourth injection (open−label phase)**										
All patients										
Elbow	81	3.5 (3.3, 3.6)	80	2.8 (2.5, 3.0)	81	2.7 (2.5, 2.9)	81	2.7 (2.5, 3.0)	81	3.0 (2.8, 3.2)
Wrist	70	3.3 (3.0, 3.5)	69	2.3 (2.1, 2.6)	70	2.3 (2.1, 2.5)	70	2.4 (2.2, 2.7)	70	3.0 (2.7, 3.2)
Fingers	77	3.5 (3.3, 3.6)	76	2.4 (2.2, 2.7)	77	2.5 (2.3, 2.7)	77	2.6 (2.4, 2.8)	77	3.1 (2.9, 3.2)
Thumb	57	3.1 (2.8, 3.3)	56	2.2 (1.9, 2.5)	57	2.2 (2.0, 2.5)	57	2.2 (2.0, 2.5)	57	2.5 (2.2, 2.8)
Shoulder[b]	56	3.2 (2.9, 3.4)	56	2.6 (2.4, 2.9)	56	2.6 (2.4, 2.9)	56	2.7 (2.5, 2.9)	56	2.9 (2.7, 3.2)
Forearm pronation[b]	46	3.4 (3.1, 3.6)	45	2.8 (2.5, 3.1)	46	2.7 (2.5, 3.0)	46	2.7 (2.5, 2.9)	46	2.9 (2.7, 3.2)

CI, confidence interval; MAS, Modified Ashworth Scale. [a] MAS scores of 0, 1, 1+, 2, 3, and 4 were coded as 0, 1, 2, 3, 4, and 5, respectively, for tabulation. [b] Injections were not delivered to the shoulder adductors/internal rotators or forearm pronators during the double−blind phase. During the open−label phase, all patients received 400 U.

Table S2. Change from baseline in DAS scores in the double−blind and open−label phases.

		Week 0	Week 2	Week 4	Week 6	Week 12
**First injection (double−blind phase)**						
400 U group	n	n/a	60	59	59	57
	Principal therapeutic target	n/a	−0.6 (−0.8, −0.4)	−0.6 (−0.8, −0.5)	−0.7 (−0.9, −0.5)	−0.6 (−0.9, −0.4)
	Hygiene	n/a	−0.3 (−0.5, −0.1)	−0.5 (−0.6, −0.3)	−0.4 (−0.6, −0.2)	−0.3 (−0.5, −0.1)
	Pain	n/a	−0.3 (−0.5, −0.1)	−0.5 (−0.7, −0.3)	−0.5 (−0.7, −0.3)	−0.4 (−0.6, −0.2)
	Dressing	n/a	−0.2 (−0.4, −0.0)	−0.4 (−0.5, −0.2)	−0.3 (−0.5, −0.1)	−0.1 (−0.3, 0.1)
	Limb position	n/a	−0.5 (−0.7, −0.3)	−0.5 (−0.7, −0.3)	−0.6 (−0.8, −0.4)	−0.4 (−0.6, −0.2)
240 U group	n	n/a	63	63	63	60
	Principal therapeutic target	n/a	−0.3 (−0.5, −0.1)	−0.4 (−0.6, −0.2)	−0.5 (−0.7, −0.3)	−0.3 (−0.5, −0.2)
	Hygiene	n/a	−0.4 (−0.6, −0.2)	−0.5 (−0.7, −0.3)	−0.5 (−0.7, −0.3)	−0.3 (−0.5, −0.1)
	Pain	n/a	−0.1 (−0.3, 0.0)	−0.3 (−0.4, −0.1)	−0.3 (−0.5, −0.2)	−0.1 (−0.3, 0.0)
	Dressing	n/a	−0.2 (−0.4, −0.1)	−0.3 (−0.4, −0.1)	−0.3 (−0.5, −0.1)	−0.2 (−0.3, −0.0)

	Limb position	n/a	−0.2 (−0.4, 0.0)	−0.3 (−0.5, −0.1)	−0.2 (−0.4, −0.0)	−0.1 (−0.3, 0.1)
**Second injection (open−label phase)**						
All patients	n	117	116	114	116	114
	Principal therapeutic target	−0.5 (−0.6, −0.3)	−0.7 (−0.8, −0.5)	−0.7 (−0.9, −0.6)	−0.7 (−0.9, −0.6)	−0.6 (−0.8, −0.5)
	Hygiene	−0.3 (−0.4, −0.2)	−0.6 (−0.7, −0.4)	−0.5 (−0.7, −0.4)	−0.6 (−0.7, −0.4)	−0.5 (−0.6, −0.3)
	Pain	−0.3 (−0.4, −0.1)	−0.3 (−0.5, −0.2)	−0.4 (−0.5, −0.2)	−0.4 (−0.5, −0.2)	−0.4 (−0.5, −0.2)
	Dressing	−0.1 (−0.3, −0.0)	−0.3 (−0.5, −0.2)	−0.4 (−0.6, −0.3)	−0.4 (−0.6, −0.3)	−0.3 (−0.4, −0.2)
	Limb position	−0.2 (−0.4, −0.1)	−0.5 (−0.7, −0.4)	−0.5 (−0.7, −0.4)	−0.6 (−0.7, −0.4)	−0.5 (−0.6, −0.4)
**Third injection (open−label phase)**						
All patients	n	111	111	110	110	110
	Principal therapeutic target	−0.6 (−0.7, −0.4)	−0.8 (−1.0, −0.6)	−0.9 (−1.0, −0.7)	−0.8 (−1.0, −0.7)	−0.7 (−0.9, −0.6)
	Hygiene	−0.5 (−0.7, −0.4)	−0.7 (−0.8, −0.5)	−0.7 (−0.9, −0.6)	−0.6 (−0.8, −0.5)	−0.5 (−0.7, −0.4)
	Pain	−0.3 (−0.5, −0.2)	−0.4 (−0.5, −0.2)	−0.5 (−0.6, −0.3)	−0.4 (−0.6, −0.3)	−0.3 (−0.5, −0.1)
	Dressing	−0.3 (−0.4, −0.2)	−0.4 (−0.5, −0.2)	−0.5 (−0.6, −0.3)	−0.5 (−0.6, −0.3)	−0.4 (−0.5, −0.3)
	Limb position	−0.4 (−0.6, −0.3)	−0.7 (−0.8, −0.5)	−0.6 (−0.8, −0.5)	−0.6 (−0.8, −0.5)	−0.5 (−0.6, −0.4)
**Fourth injection (open−label phase)**						
All patients	n	83	82	83	83	83
	Principal therapeutic target	−0.7 (−0.9, −0.5)	−0.8 (−1.0, −0.6)	−0.8 (−1.0, −0.6)	−0.8 (−1.0, −0.6)	−0.8 (−1.0, −0.6)
	Hygiene	−0.5 (−0.6, −0.3)	−0.6 (−0.8, −0.4)	−0.6 (−0.8, −0.4)	−0.6 (−0.8, −0.4)	−0.6 (−0.8, −0.5)
	Pain	−0.2 (−0.4, −0.0)	−0.3 (−0.5, −0.1)	−0.3 (−0.5, −0.1)	−0.3 (−0.5, −0.1)	−0.4 (−0.6, −0.2)
	Dressing	−0.4 (−0.5, −0.2)	−0.4 (−0.6, −0.3)	−0.4 (−0.6, −0.3)	−0.4 (−0.6, −0.2)	−0.4 (−0.6, −0.3)
	Limb position	−0.5 (−0.6, −0.3)	−0.6 (−0.7, −0.4)	−0.6 (−0.8, −0.4)	−0.6 (−0.8, −0.4)	−0.6 (−0.7, −0.4)

DAS, Disability Assessment Scale; n/a, not applicable. Mean (95% confidence interval). Negative values indicate an improvement from baseline. During the open−label phase, all patients received 400 U.

Table S3. CGI in the double−blind and open−label phases.

		Week 2		Week 4		Week 6		Week 12	
		n	Mean (95% CI)	n	Mean (95% CI)	n	Mean (95% CI)	n	Mean (95% CI)
**First injection (double−blind phase)**									
Physician−assessed CGI	400 U group	60	1.8 (1.6, 2.1)	59	1.8 (1.6, 2.1)	59	1.5 (1.3, 1.8)	57	0.9 (0.6, 1.2)
	240 U group	63	1.6 (1.3, 1.8)	63	1.6 (1.4, 1.9)	63	1.4 (1.1, 1.7)	60	0.6 (0.3, 1.0)
Patient−assessed CGI	400 U group	60	1.6 (1.3, 1.8)	59	1.5 (1.2, 1.8)	59	1.2 (0.9, 1.4)	57	0.6 (0.3, 0.9)
	240 U group	63	1.4 (1.2, 1.7)	63	1.4 (1.1, 1.7)	63	1.3 (1.0, 1.6)	60	0.7 (0.4, 1.0)

**Second injection (open−label phase)**									
Physician−assessed CGI	400 U group	56	1.9 (1.7, 2.1)	56	1.9 (1.6, 2.1)	57	1.5 (1.2, 1.8)	57	1.0 (0.7, 1.3)
	240 U group	60	2.1 (1.8, 2.3)	58	2.0 (1.7, 2.2)	59	1.8 (1.6, 2.1)	57	0.9 (0.6, 1.3)
Patient−assessed CGI	400 U group	56	1.4 (1.1, 1.7)	56	1.4 (1.2, 1.7)	57	1.3 (1.0, 1.6)	57	0.7 (0.4, 1.0)
	240 U group	60	1.9 (1.6, 2.2)	58	1.8 (1.6, 2.1)	59	1.6 (1.3, 1.9)	57	1.0 (0.7, 1.4)
**Third injection (open−label phase)**									
Physician−assessed CGI	400 U group	56	1.9 (1.6, 2.2)	56	1.6 (1.3, 1.9)	55	1.5 (1.3, 1.8)	55	0.9 (0.6, 1.3)
	240 U group	55	1.8 (1.5, 2.1)	54	1.7 (1.4, 1.9)	55	1.3 (1.0, 1.6)	55	0.8 (0.5, 1.1)
Patient−assessed CGI	400 U group	56	1.5 (1.2, 1.8)	56	1.4 (1.1, 1.7)	55	1.1 (0.9, 1.4)	55	0.8 (0.5, 1.1)
	240 U group	55	1.7 (1.4, 2.0)	54	1.6 (1.3, 1.9)	55	1.4 (1.1, 1.7)	55	0.9 (0.6, 1.2)
**Fourth injection (open−label phase)**									
Physician−assessed CGI	400 U group	39	1.8 (1.6, 2.1)	40	1.4 (1.0, 1.8)	40	1.4 (1.1, 1.7)	40	1.1 (0.7, 1.5)
	240 U group	43	1.7 (1.4, 2.0)	43	1.5 (1.2, 1.8)	43	1.5 (1.1, 1.8)	43	1.3 (0.9, 1.7)
Patient−assessed CGI	400 U group	39	1.3 (1.0, 1.6)	40	1.2 (0.8, 1.5)	40	1.1 (0.7, 1.4)	40	0.8 (0.5, 1.1)
	240 U group	43	1.4 (1.1, 1.7)	43	1.5 (1.1, 1.8)	43	1.2 (0.9, 1.5)	43	0.9 (0.5, 1.3)

CGI, Clinical Global Impression of Change; CI, confidence interval. Positive values indicate an improvement from baseline. During the open−label phase, all patients received 400 U.

Table S4. Common adverse events during the overall study period (48 weeks).

	400 U group (N = 61)	240 U group (N = 63)
Total number of patients with adverse events	49 (80%)	52 (83%)
Fall	15 (25%)	11 (17%)
Nasopharyngitis	10 (16%)	15 (24%)
Contusion	9 (15%)	6 (10%)
Seasonal allergy	5 (8%)	2 (3%)
Constipation	5 (8%)	1 (2%)
Insomnia	4 (7%)	1 (2%)
Back pain	3 (5%)	6 (10%)
Oxygen saturation decreased	3 (5%)	5 (8%)
Arthralgia	3 (5%)	2 (3%)
Musculoskeletal pain	3 (5%)	2 (3%)
Dental caries	3 (5%)	2 (3%)
Hypertension	2 (3%)	4 (6%)
Influenza	2 (3%)	3 (5%)
Gingivitis	2 (3%)	2 (3%)
Pharyngitis	2 (3%)	2 (3%)
Upper respiratory tract infection	2 (3%)	2 (3%)
Skin abrasion	2 (3%)	2 (3%)
Pneumonia	2 (3%)	1 (2%)
Tinea pedis	2 (3%)	1 (2%)
Pain in extremity	2 (3%)	1 (2%)
Eczema	2 (3%)	1 (2%)
Dermatophytosis of nail	2 (3%)	0
Sinusitis	2 (3%)	0
Muscle spasms	2 (3%)	0
Cerebral hemorrhage	2 (3%)	0
Parkinsonism	2 (3%)	0

Hemorrhage subcutaneous	2 (3%)	0
Hyperkeratosis	2 (3%)	0
Pollakiuria	2 (3%)	0
Ligament sprain	1 (2%)	3 (5%)
Pyrexia	0	3 (5%)
Bronchitis	0	2 (3%)
Rib fracture	0	2 (3%)
Toothache	0	2 (3%)
Alanine aminotransferase increased	0	2 (3%)
Erythema	0	2 (3%)

Number of patients (%). Adverse events reported by more than one patient in either group are shown. Note that all patients received 400 U during the open−label phase.

Table S5. Joints treated in the open−label phase (117 patients, 311 treatment sessions).

Joints	No. of patients	No. of treatment sessions	Mean dose per session	95% CI	Between−patient standard deviation	Within−patient standard deviation
Shoulder	88	206	76.4	(67.7, 85.2)	38.38	21.84
Elbow	117	306	132.9	(126.2, 139.6)	31.63	29.07
Forearm (pronation)	73	175	49.2	(45.0, 53.4)	16.28	11.46
Wrist	109	270	82.4	(77.4, 87.4)	22.94	19.32
Fingers	114	291	92.5	(86.4, 98.7)	31.07	17.77
Thumb	105	227	42.3	(39.2, 45.4)	13.33	12.71

CI, confidence interval.

Table S6. Muscles injected in the open−label phase (117 patients, 311 treatment sessions).

Muscles	No. of patients	No. of treatment sessions	Mean dose per session	95% CI	Between−patient standard deviation	Within−patient standard deviation
Pectoralis major	84	195	56.2	(51.5, 60.8)	19.59	12.78
Latissimus dorsi	36	69	45.8	(38.4, 53.1)	19.76	11.60
Teres major	16	27	42.1	(34.9, 49.3)	12.48	6.04
Subscapularis	9	17	42.7	(33.9, 51.4)	10.34	6.15
Biceps brachii	116	296	74.0	(70.4, 77.6)	16.42	17.08
Brachialis	110	261	45.3	(42.6, 47.9)	12.82	8.53
Brachioradialis	87	168	41.6	(38.9, 44.2)	11.07	7.29
Pronator teres	73	175	47.2	(43.9, 50.5)	12.57	9.66
Pronator quadratus	4	8	45.3	(15.4, 75.2)	17.59	8.71
Flexor carpi radialis	108	267	50.1	(46.9, 53.2)	14.79	10.62
Flexor carpi ulnaris	94	213	42.4	(40.1, 44.6)	9.58	7.69
Flexor digitorum profundus	85	200	48.8	(45.0, 52.6)	16.88	7.09
Flexor digitorum superficialis	111	276	56.2	(52.9, 59.5)	16.15	10.37
Lumbricals	22	46	39.6	(31.1, 48.1)	18.74	5.62
Flexor pollicis longus	84	174	28.5	(26.1, 30.9)	10.48	4.30
Adductor pollicis	80	166	28.9	(26.0, 31.8)	12.65	4.34
Opponens pollicis	7	12	–	–	8.08	0.00

CI, confidence interval. Some estimates were not obtained because of the small amount of data.

Table S7. Muscles injected to treat shoulder adduction/internal rotation (88 patients, 206 treatment sessions).

Muscles	No. of patients	No. of treatment sessions	Crude mean rate (%)[a]	Adjusted mean rate (%)[b]	95% CI	Mean dose per session	95% CI	Mean dose per muscle			
								PM	LD	TM	SS
PM	58	111	35.7	35.5	(28.4, 44.4)	55.3	(50.7, 60.0)	55.3	–	–	–
PM + LD	30	54	17.4	17.4	(11.9, 25.7)	101.0	(84.8, 117.1)	55.4	45.5	–	–
PM + TM	6	10	3.2	3.2	(1.2, 8.3)	–	–	–	–	–	–
PM + LD + TM	5	8	2.6	2.7	(0.9, 8.1)	146.0	(67.5, 224.4)	56.7	50.6	–	–
TM	4	4	1.3	1.3	(0.5, 3.4)	–	–	–	–	–	–
SS	3	4	1.3	1.5	(0.4, 6.1)	43.8	(16.9, 70.6)	–	–	–	43.8
PM + SS	2	4	1.3	1.2	(0.2, 6.9)	90.3	(−384.9, 565.5)	54.2	–	–	–
PM + LD + SS	2	4	1.3	1.2	(0.2, 6.9)	–	–	–	–	–	–
PM + TM + SS	2	3	1.0	0.9	(0.2, 4.5)	170.8	(−65.9, 407.6)	83.3	–	–	–
LD	2	2	0.6	–	–	–	–	–	–	–	–
TM + SS	1	1	0.3	0.3	(0, 2.3)	–	–	–	–	–	–
PM + LD + TM + SS	1	1	0.3	0.3	(0, 2.3)	–	–	–	–	–	–

CI, confidence interval; LD, latissimus dorsi; PM, pectoralis major; SS, subscapularis; TM, teres major. [a]Percentages are based on the number of all treatment sessions (311) in the open−label phase. [b]Adjusted mean rates were estimated using negative binomial distribution. Some estimates were not obtained because of the small amount of data.

Table S8. Muscles injected to treat elbow flexion (117 patients, 306 treatment sessions).

Muscles	No. of patients	No. of treatment sessions	Crude mean rate (%)[a]	Adjusted mean rate (%)[b]	95% CI	Mean dose per session	95% CI	Mean dose per muscle		
								BB	B	BR
BB+B+BR	74	147	47.3	47.3	(39.8, 56.3)	160.2	(151.8, 168.6)	73.4	46.3	40.6
BB+B	58	106	34.1	33.9	(27.4, 42.0)	117.7	(109.7, 125.6)	71.3	46.3	–
BB	25	28	9.0	9.0	(6.2, 13.0)	89.6	(81.2, 98.1)	89.6	–	–
BB+BR	12	15	4.8	4.8	(2.6, 8.6)	125.0	(101.7, 148.4)	78.3	–	47.2
B	4	4	1.3	1.3	(0.5, 3.4)	–	–	–	–	–
B+BR	2	4	1.3	1.4	(0.2, 9.1)	–	–	–	–	–
BR	2	2	0.6	0.6	(0.2, 2.6)	–	–	–	–	–

B, brachialis; BB, biceps brachii; BR, brachioradialis; CI, confidence interval. [a]Percentages are based on the number of all treatment sessions (311) in the open−label phase. [b]Adjusted mean rates were estimated using negative binomial distribution. Some estimates were not obtained because of the small amount of data.

Table S9. Muscles injected to treat forearm pronation (73 patients, 175 treatment sessions).

Muscles	No. of patients	No. of treatment sessions	Crude mean rate (%)[a]	Adjusted mean rate (%)[b]	95% CI	Mean dose per session	95% CI	Mean dose per muscle	
								PT	PQ
PT	71	167	53.7	53.7	(45.5, 63.3)	47.1	(43.8, 50.5)	47.1	–
PT+PQ	4	8	2.6	2.5	(0.7, 8.6)	99.4	(40.4, 158.4)	54.2	45.3

CI, confidence interval; PQ, pronator quadratus; PT, pronator teres. [a]Percentages are based on the number of all treatment sessions (311) in the open−label phase. [b]Adjusted mean rates were estimated using negative binomial distribution. Some estimates were not obtained because of the small amount of data.

Table S10. Muscles injected to treat wrist flexion (109 patients, 270 treatment sessions).

Muscles	No. of patients	No. of treatment sessions	Crude mean rate (%)[a]	Adjusted mean rate (%)[b]	95% CI	Mean dose per session	95% CI	Mean dose per muscle	
								FCR	FCU
FCR+FCU	93	210	67.5	67.5	(59.0, 77.3)	91.2	(86.0, 96.5)	48.9	42.3
FCR	36	57	18.3	18.1	(13.1, 25.1)	53.7	(50.2, 57.2)	53.7	–
FCU	3	3	1.0	–	–	–	–	–	–

CI, confidence interval; FCR, flexor carpi radialis; FCU, flexor carpi ulnaris. [a]Percentages are based on the number of all treatment sessions (311) in the open–label phase. [b]Adjusted mean rates were estimated using negative binomial distribution. Some estimates were not obtained because of the small amount of data.

Table S11. Muscles injected to treat finger flexion (114 patients, 291 treatment sessions).

Muscles	No. of patients	No. of treatment sessions	Crude mean rate (%)[a]	Adjusted mean rate (%)[b]	95% CI	Mean dose per session	95% CI	Mean dose per muscle		
								FDP	FDS	LI
FDP+FDS	75	177	56.9	56.9	(48.8, 66.4)	102.9	(95.9, 109.9)	49.2	53.6	–
FDS	37	66	21.2	21.3	(15.3, 29.8)	60.7	(53.8, 67.5)	–	60.7	–
FDP+FDS+LI	10	18	5.8	5.8	(2.7, 12.5)	136.6	(118.4, 154.9)	41.1	63.3	–
FDS+LI	9	15	4.8	4.6	(2.2, 9.9)	110.3	(92.5, 128.1)	–	67.9	42.3
LI	6	10	3.2	3.2	(1.2, 8.3)	–	–	–	–	–
FDP	2	2	0.6	–	–	75.0	(−242.7, 392.7)	75.0	–	–
FDP+LI	1	3	1.0	0.9	(0.1, 14.5)	91.7	–	66.7	–	–

CI, confidence interval; FDP, flexor digitorum profundus; FDS, flexor digitorum superficialis; LI, lumbricals. [a]Percentages are based on the number of all treatment sessions (311) in the open–label phase. [b]Adjusted mean rates were estimated using negative binomial distribution. Some estimates were not obtained because of the small amount of data.

Table S12. Muscles injected to treat thumb flexion/adduction (105 patients, 227 treatment sessions).

Muscles	No. of patients	No. of treatment sessions	Crude mean rate (%)[a]	Adjusted mean rate (%)[b]	95% CI	Mean dose per session	95% CI	Mean dose per muscle		
								FPL	AP	OP
FPL+AP	55	111	35.7	36.0	(28.3, 45.9)	52.2	(46.9, 57.6)	27.2	25.0	–
FPL	38	56	18.0	18.0	(13.3, 24.5)	30.6	(26.9, 34.4)	30.6	–	–
AP	33	48	15.4	15.5	(11.1, 21.7)	37.8	(32.5, 43.0)	–	37.8	–
FPL+OP	3	4	1.3	1.4	(0.4, 5.0)	–	–	–	–	–
AP+OP	2	4	1.3	1.2	(0.2, 6.9)	–	–	–	–	–
FPL+AP+OP	2	3	1.0	0.9	(0.2, 4.5)	–	–	–	–	–
OP	1	1	0.3	–	–	–	–	–	–	–

AP, adductor pollicis; CI, confidence interval; FPL, flexor pollicis longus; OP, opponens pollicis. [a]Percentages are based on the number of all treatment sessions (311) in the open–label phase. [b]Adjusted mean rates were estimated using negative binomial distribution. Some estimates were not obtained because of the small amount of data

Table S13. Mean doses per muscle separated by the MAS score prior to injection.

Joints	Muscles	No. of patients	MAS before injection	Adjusted mean	95% CI	Difference from MAS 0–2	95% CI
Shoulder	Pectoralis major	84	3 or 4	58.9	(53.4, 64.5)	5.1	(−0.7, 11.0)

			0 to 2	53.8	(48.5, 59.1)		
	Latissimus dorsi	36	3 or 4	49.9	(40.6, 59.1)	7.9	(−3.1, 18.8)
			0 to 2	42.0	(33.1, 51.0)		
	Teres major	16	3 or 4	37.2	(28.1, 46.2)	−8.4	(−17.8, 1.1)
			0 to 2	45.5	(37.4, 53.6)		
	Subscapularis	9	3 or 4	45.6	(35.5, 55.6)	5.2	(−3.2, 13.6)
			0 to 2	40.4	(30.7, 50.1)		
Elbow	Biceps brachii	116	3 or 4	75.2	(70.9, 79.5)	2.7	(−2.6, 8.1)
			0 to 2	72.5	(67.7, 77.2)		
	Brachialis	110	3 or 4	46.3	(43.3, 49.2)	2.3	(−0.9, 5.6)
			0 to 2	43.9	(40.7, 47.2)		
	Brachioradialis	87	3 or 4	43.1	(40.1, 46.1)	4.0	(0.5, 7.5)
			0 to 2	39.2	(35.8, 42.5)		
Forearm	Pronator teres	73	3 or 4	50.9	(47.2, 54.5)	7.6	(3.6, 11.5)
			0 to 2	43.3	(39.6, 47.0)		
	Pronator quadratus	4	3 or 4	–	–	–	–
			0 to 2	–	–		
Wrist	Flexor carpi radialis	108	3 or 4	54.7	(51.1, 58.3)	8.2	(4.3, 12.1)
			0 to 2	46.5	(43.2, 49.9)		
	Flexor carpi ulnaris	94	3 or 4	46.1	(43.4, 48.8)	6.7	(3.6, 9.8)
			0 to 2	39.4	(36.9, 41.9)		
Fingers	Flexor digitorum profundus	85	3 or 4	50.3	(46.0, 54.6)	2.5	(−1.0, 6.1)
			0 to 2	47.8	(43.8, 51.8)		
	Flexor digitorum superficialis	111	3 or 4	59.0	(55.1, 62.9)	4.7	(1.0, 8.5)
			0 to 2	54.3	(50.7, 57.8)		
	Lumbricals	22	3 or 4	39.2	(29.6, 48.8)	−0.6	(−6.9, 5.8)
			0 to 2	39.8	(31.0, 48.5)		
Thumb	Flexor pollicis longus	84	3 or 4	30.4	(27.4, 33.4)	2.7	(−0.1, 5.5)
			0 to 2	27.7	(25.2, 30.1)		
	Adductor pollicis	80	3 or 4	31.7	(28.3, 35.0)	4.2	(1.4, 7.0)
			0 to 2	27.5	(24.6, 30.4)		
	Opponens pollicis	7	3 or 4	–	–	–	–
			0 to 2	–	–		

CI, confidence interval; MAS, Modified Ashworth Scale. Some estimates were not obtained because of the small amount of data.
